# Automated craniofacial landmarks detection on 3D image using geometry characteristics information

**DOI:** 10.1186/s12859-018-2548-9

**Published:** 2019-02-04

**Authors:** Arpah Abu, Chee Guan Ngo, Nur Idayu Adira Abu-Hassan, Siti Adibah Othman

**Affiliations:** 1 Institute of Biological Sciences, Faculty of Science, University of Malaya, 50603 Kuala Lumpur, Malaysia; 2 Centre of Research for Computational Sciences and Informatics for Biology, Bioindustry, Environment, Agriculture and Healthcare, University of Malaya, 50603 Kuala Lumpur, Malaysia; 3 Department of Paediatric Dentistry and Orthodontics / Clinical Craniofacial Dentistry Research Group, Faculty of Dentistry, University of Malaya, 50603 Kuala Lumpur, Malaysia; 4 Department of Diagnostic & Allied Health Science, Faculty of Health & Life Sciences, Management & Science University, Shah Alam, Selangor Malaysia

**Keywords:** Indirect anthropometry, Automated craniofacial landmarks, 3D facial image, Geometry characteristics information

## Abstract

**Background:**

Indirect anthropometry (IA) is one of the craniofacial anthropometry methods to perform the measurements on the digital facial images. In order to get the linear measurements, a few definable points on the structures of individual facial images have to be plotted as landmark points. Currently, most anthropometric studies use landmark points that are manually plotted on a 3D facial image by the examiner. This method is time-consuming and leads to human biases, which will vary from intra-examiners to inter-examiners when involving large data sets. Biased judgment also leads to a wider gap in measurement error. Thus, this work aims to automate the process of landmarks detection to help in enhancing the accuracy of measurement. In this work, automated craniofacial landmarks (ACL) on a 3D facial image system was developed using geometry characteristics information to identify the nasion (*n*), pronasale (*prn*), subnasale (*sn*), alare (*al*), labiale superius (*ls*), stomion (*sto*), labiale inferius (*li*), and chelion (*ch*). These landmarks were detected on the 3D facial image in .obj file format. The IA was also performed by manually plotting the craniofacial landmarks using Mirror software. In both methods, once all landmarks were detected, the eight linear measurements were then extracted. Paired t-test was performed to check the validity of ACL (i) between the subjects and (ii) between the two methods, by comparing the linear measurements extracted from both ACL and AI. The tests were performed on 60 subjects (30 males and 30 females).

**Results:**

The results on the validity of the ACL against IA between the subjects show accurate detection of *n*, *sn*, *prn*, *sto*, *ls* and *li* landmarks. The paired t-test showed that the seven linear measurements were statistically significant when *p* < 0.05. As for the results on the validity of the ACL against IA between the methods, ACL is more accurate when p ≈ 0.03.

**Conclusions:**

In conclusion, ACL has been validated with the eight landmarks and is suitable for automated facial recognition. ACL has proved its validity and demonstrated the practicability to be used as an alternative for IA, as it is time-saving and free from human biases.

## Background

Craniofacial anthropometry is the science of measuring the human face and head [1]. It provides a simple and non-invasive quantitative assessment method to assess the surface changes of the anatomy of human faces. There are two methods of craniofacial anthropometry, which are direct anthropometry and indirect anthropometry.

Farkas [2] categorized the act of measurement with the need for physical contact with the subject as direct anthropometry. This manual measurement of the physical examination is a tiring process because it is time-consuming, and greatly dependent on the cooperation of the subject and the skills of the clinician. For instance, craniofacial anthropometry has been used in analyzing Down syndrome patients [3], in which direct anthropometry was performed on 104 Caucasian patients by a measurer to obtain 25 craniofacial measurements per patient. Twenty to 30 min were taken to complete the measurement for just one cooperative patient. Fakhroddin et al. [4] performed a study on craniofacial measurement of 68 male and 33 female patients with chronic schizophrenia (based on DSM-IV criteria), and 50 male and 51 female healthy volunteers. They took less time to complete the measurement for one subject. However, measurements were performed by two people to further ensure its accuracy. In other examples such as the anthropometric study on the patterns of dysmorphology in Crouzon syndrome [5] and the surface morphology in Treacher Collins syndrome [6], anthropometric measurements were obtained from 61 patients and 18 patients respectively. All measurements were taken by Farkas alone to ensure the consistency of the results. However, not all measurements were taken into account due to the lack of cooperation among some patients.

Thus, an indirect anthropometry method was then introduced to overcome the direct method, whereby 2D photographs or 3D images of the human face were captured using a camera imaging system and the measurement was performed on the 2D photographs or 3D images. Most of the works in indirect anthropometry use landmark points that are manually plotted on the photograph or 3D image. For instance, Edler et al. [7] studied facial attractiveness using 15 facial photographs of orthoganthic patients which were taken by the same medical photographer and the landmarks were marked manually on the photographs. In a research on a 3D head anthropometric analysis [8], the 3D images were acquired by using Eyetronics, a light-based imaging system which consists of a regular slide projector, a digital camera, and a calibration pattern. Landmarks were pre-labelled on a mannequin head by placing a small triangle, red-colored paper with a blue dot on the landmarks’ location. Imaging was performed on the mannequin head and the landmarks’ coordinates were retrieved. Measurements were computed using 3D Euclidian distance between landmarks after anthropometric landmarks were identified and localized. Still, this method is time-consuming and leads to human bias, which will vary within the intra-examiner himself and among inter-examiners when involving large data sets. Biased judgment leads to a wider range of measurement error, and is time-consuming because more time is needed to plot the landmarks.

In summary, both methods share some similarities and differences. Both methods require well-trained personnel to perform the measurements. The accuracy of the measurements may differ among examiners when a large dataset is involved. Both methods are time-consuming when dealing with patients and plotting the landmarks.

Despite that, indirect anthropometry is better than direct anthropometry whereby the facial image is captured using a camera imaging system followed by performing the measurement on the captured image. It is also more convenient in comparison to direct anthropometry. Once the facial image has been captured, measurements can be performed by anyone at any time. As for the case of direct anthropometry, an appointment has to be made between the operator and patient to carry out the measurement. Re-measurement is impossible without the patients being present.

Since plotting the landmarks manually is a labor-intensive process, several automated systems have been developed to locate feature points on 2D, 2.5D, and 3D images. 2D images are able to visualize width and height while 3D images are able to visualize width, height, and additional depth information. A 2.5D image is an image where only one depth value is provided. In 2D images, the intensity or color information is analyzed. In contrast, geometric characteristics information is analyzed in 2.5D or 3D images. Facial feature detection systems can be solely dependent on geometry characteristics information or further supported by various statistical models. As 3D images acquisition systems have become popular and mature, the database of 3D human facial images [9, 10] has increased tremendously.

Beumer et al. [11] explored and compared two methods in detecting the landmarks, the Most Likely-Landmark Locator (MLLL) which is based on maximizing the likelihood ratio and Viola-Jones detection [12] which uses a combination of Haar-like features to represent the texture information in an image. Another method is the Active Appearance Models (AAM), as proposed by Cootes et al. [13], which uses a joint statistical model of appearance and shape.

Furthermore, Gökberk et al. [14] proposed an average face model for detecting the landmarks on a 3D image. All landmarks are predefined on the average face model. Landmarks registration is done by aligning the 3D human facial image with the average face model and using an iterative closest point algorithm. After that, fine tuning is done by using shape descriptors such as mean curvature, Gaussian curvature, and others. Alternatively, Nair and Cavallaro [15] used a 3D facial model based on a Point Distribution Model (PDM) to represent the shape of the region of interest that includes the required landmarks, along with the statistical information of the shape variation across the training set.

Guo et al. [16] proposed a system that starts with a set of raw 3D face scans wherein the nose tip is first localized using a sphere fitting approach. Subsequently, pose normalization is performed to align a sample face for a uniform frontal view. Six of the most salient landmarks are first manually plotted on a set of training samples. Then, Principal Component Analysis (PCA) is performed to localize these six landmarks on the sample surfaces and the 11 additional landmarks are heuristically annotated afterwards. A reference face is chosen and re-meshed using spherical sampling, then TPS-warped to each sample face using the 17 landmark points. A dense, biological correspondence is built by re-meshing the sample face according to the reference face. The correspondence is further improved by using an average face model as the reference and repeating the registration process.

Furthermore, Liang et al. [17] proposed a method to locate 27 landmarks on a 3D mesh. In this study, the 17 geometrically-determined landmarks on the individual 3D meshes were used in the initial correspondence required by the deformable matching. To improve the accuracy and produce 20 landmarks that are globally accepted, a deformable matching procedure establishes a dense correspondence from a template 3D mesh with a full set of 20 landmarks to each individual 3D mesh.

Hence, the current available systems are still said to have many limitations, particularly in domain-specific applications, such as facial image retrieval [18]. This work aims to improve upon the previous work limitations in term of recognition accuracy, dimensionality reduction, and implementing a 3D environment as a full foundation for craniofacial analysis instead of a 2D environment. Most of the previous works which fully utilize the 2D images, such as [11–13], make little or no attempt to maximize sources of 3D facial data in their study sample and employ it in their respective methods.

The objectives of this study are: (1) to detect the landmark locations using geometry characteristics information; (2) to extract the linear measurements using 3D Euclidean distance functions; (3) to compute the proportional indices from the ratio of linear measurements; and (4) to evaluate the system by performing the validity testing using paired test. In addition, a graphical user interface is developed as a prototype for the end user system.

## Methods

### Study design

**Fig. 1:**
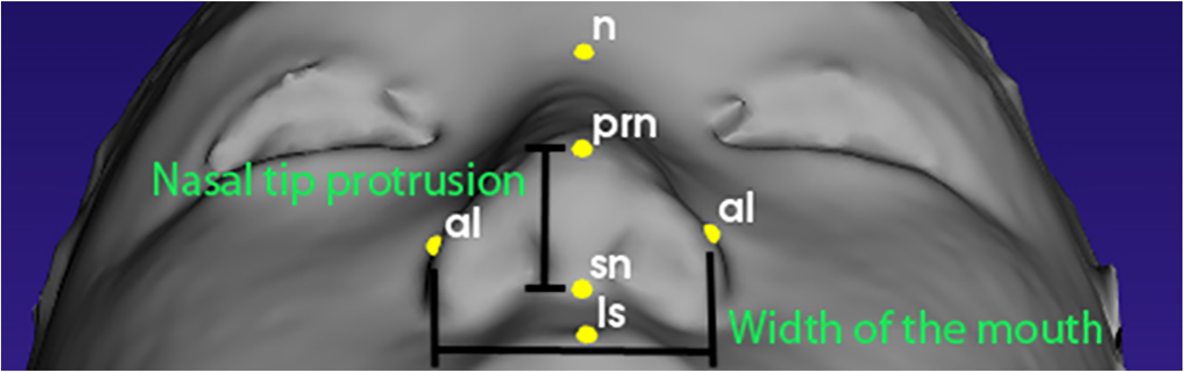
Nose region

**Fig. 2:**
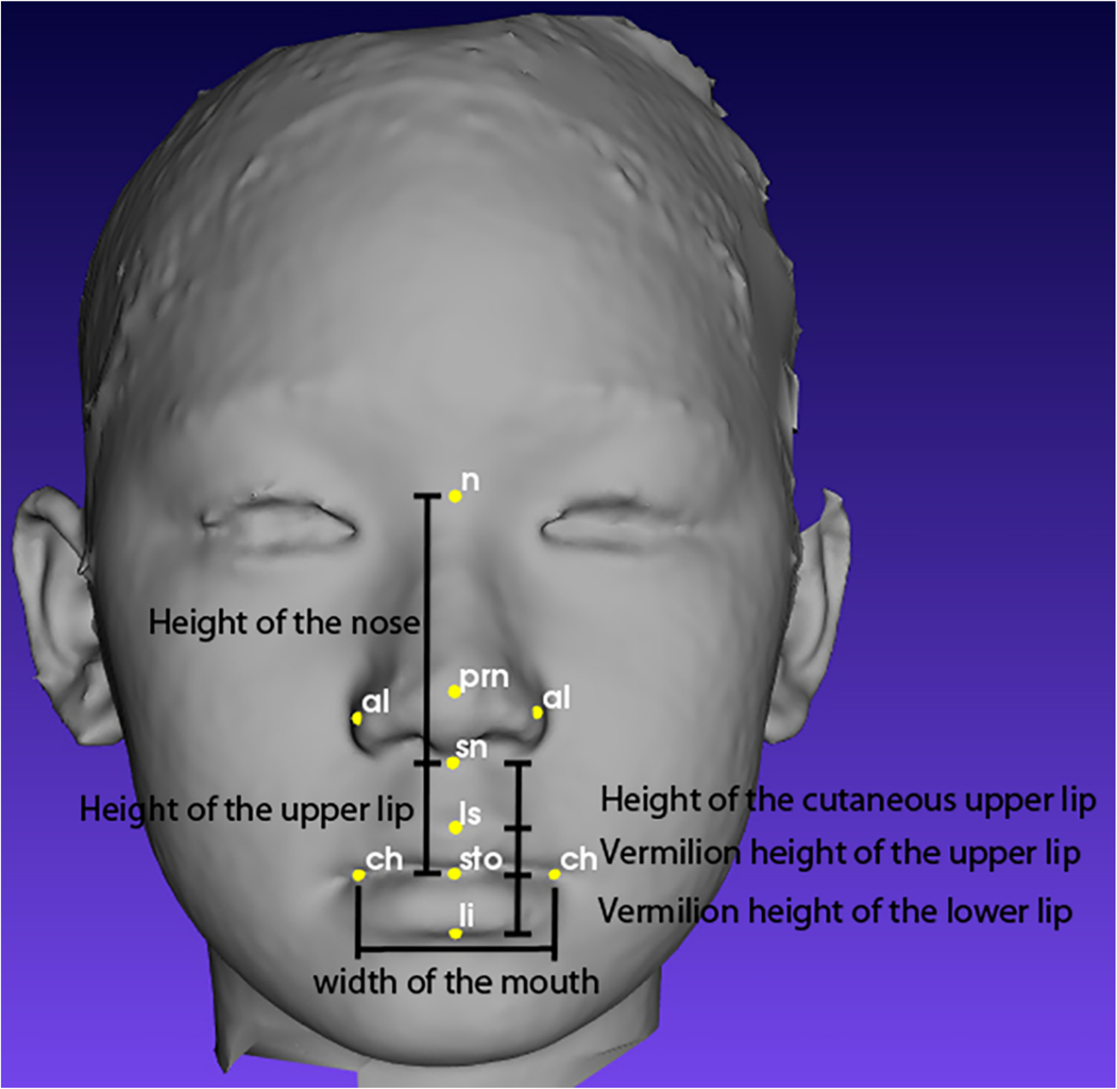
Orolabial region

As mentioned in Othman et al. [19], there are four regions of the craniofacial complex – face, orbits, nose, and orolabial areas with 18 facial landmarks. However, in this work, only ten landmarks, namely nasion (*n*), pronasale (*prn*), subnasale (*sn*), alare (*al*) for both left and right, labiale superius (*ls*), stomion (*sto*), labiale inferius (*li*), and chelion (*ch*) for both left and right, which represents the nose and orolabial regions were located on the 3D facial image as shown in Figs. 1 and 2.

Once all ten landmarks were detected, eight linear measurements were acquired for height of the nose (*n-sn*), width of the nose (*alL-alR*), nasal tip protrusion (*sn-prn*), width of the mouth (*chL-chR*), height of the upper lip (*sn-sto*), vermilion height of the upper lip (*ls-sto*), height of the cutaneous upper lip (*sn-ls*), and vermilion height of the lower lip (*sto-li*) using the Euclidean distance functions as shown in Table 1. Proportional indices were then calculated for nasal index, nasal tip protrusion width index, upper lip width index, skin portion upper lip index, and upper vermilion height index as shown in Table 2.

**Table 1:** Linear measurements extracted from the nose and orolabial regions

Region	Linear Measurements	Euclidean distance between
Nose	Height of the nose	nasion (*n*) and subnasale (*sn*)
	Width of the nose	alare (*al*)
	Nasal tip protrusion	subnasale (*sn*) and pronasale (*prn*)
Orolabial	Width of the mouth	chelion (*ch*)
	Height of the upper lip	subnasale (*sn*) and stomion (*sto*)
	Vermilion height of the upper lip	labiale superius (*ls*) and stomion (*sto*)
	Height of the cutaneous upper lip	subnasale (*sn*) and labiale superius (*ls*)
	Vermilion height of the lower lip	stomion (*sto*) and labiale inferius (*li*)

**Table 2:** Proportional indices extracted from the nose and orolabial regions

Regions	Proportional Indices
Nose	$Nasal\ index=\frac{Width\ of\ the\ nose}{Height\ of\ the\ nose}\times 100$
	$$ Nasal\ tip\ protrusion\ width\ index=\frac{Nasal\ tip\ protrusion}{Width\ of\ the\ nose}\times 100 $$
Orolabial	$Upper\ lip\ width\ index=\frac{Height\ of\ the\ upper\ lip}{Width\ of\ the\ mouth}\times 100$
	$$ Skin\ portion\ upper\ lip\ index=\frac{Height\ of\ the\ cutaneous\ upper\ lip}{Height\ of\ the\ upper\ lip}\times 100 $$
	$$ Upper\ vermilion\ height\ index=\frac{Vermilion\ height\ of\ the\ upper\ lip}{Vermilion\ height\ of\ the\ lower\ lip}\times 100 $$

The 3D facial images of 30 female subjects and 30 male subjects, aged between 18 to 25 years old were imported into the designed system for detecting the craniofacial landmarks automatically as well as for plotting the landmarks manually by the examiners in the indirect anthropometry method.

The ethical and written approval for this study was obtained from the University of Malaya Medical Ethics Committee [DF CD 1211/0059(L)]. All subjects were given verbal and written explanations regarding the study to obtain consent. Written informed consent was also obtained from each patient for the publication of this report and any accompanying images.

### Subjects and imaging system

A database of raw 3D facial images, acquired from a 3D stereophotogrammetry system during data acquisition, was used as the data sample. The 3D facial images were obtained by using a stereophotogrammetry camera which is available at the 3D Imaging Lab, Department of Paediatric Dentistry and Orthodontics, Faculty of Dentistry, University of Malaya.

**Fig. 3:**
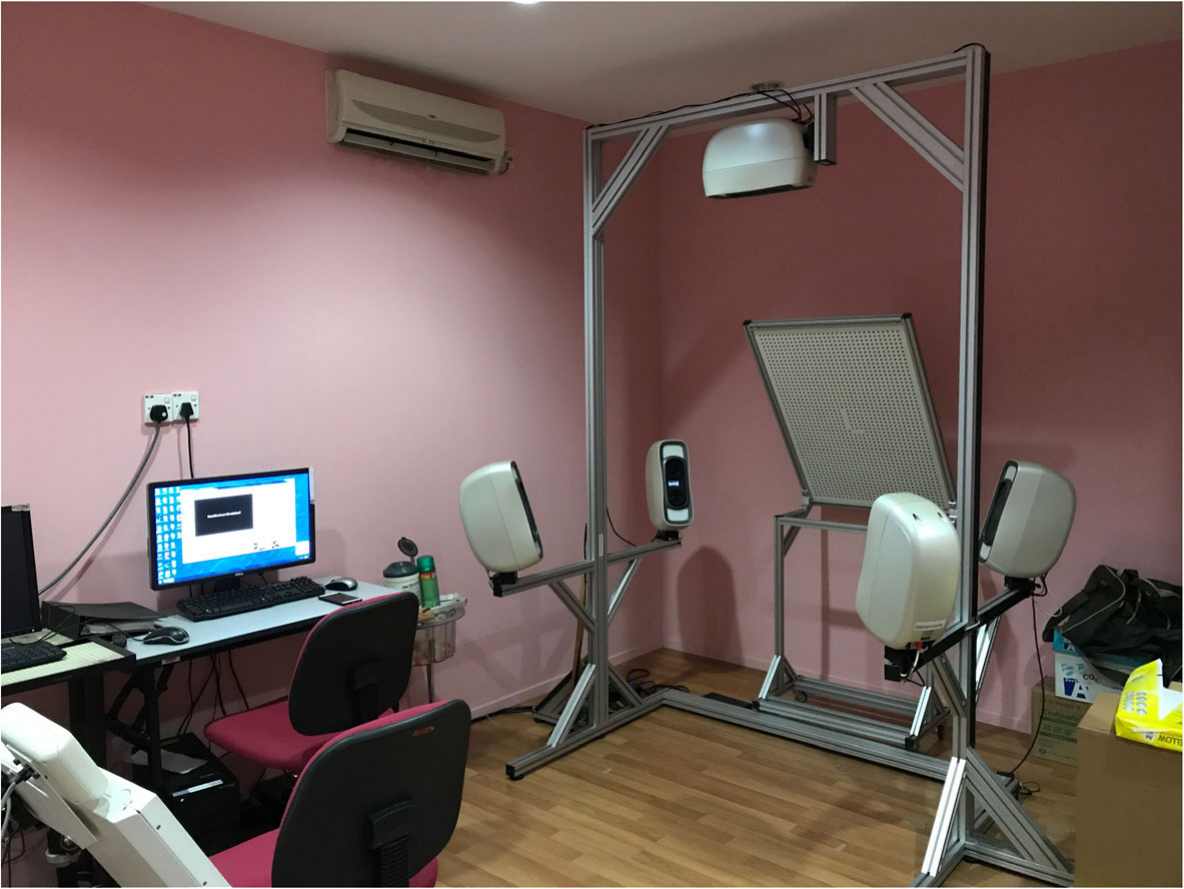
The Vectra-M5 360 camera imaging system

The Vectra-M5 360 (Canfield Scientific Inc., Fairfield NJ, USA) System, as shown in Fig. 3, is a three-dimensional stereophotogrammetry camera system which consists of five cameras. The camera was calibrated according to the manufacturer’s guidelines before using. Subjects were seated and positioned at the center of the camera system. They were required to wear a head cap in order to cover their hair. For males, shaving was required as the camera is unable to retexture any facial hair.

This set of cameras was linked to a desktop computer with a dedicated software known as Mirror software. Generally, Mirror software is used for 3D image capturing, mapping and processing to view the facial images, annotating the landmarks manually, measuring the distance, and other image simulations.

**Fig. 4:**
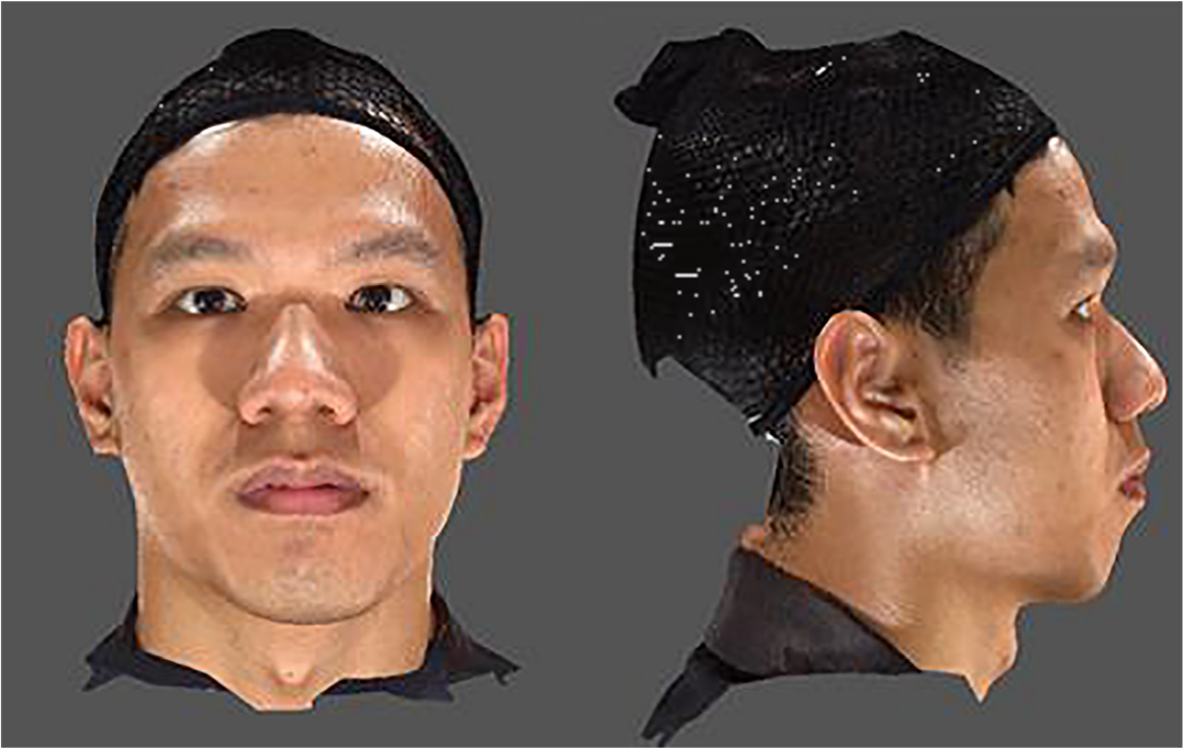
Raw 3D facial image

As shown below, Fig. 4 is a raw, unprocessed 3D facial image of the front view and profile view, without any landmarks annotated.

### Automated craniofacial landmarks (ACL) using geometry characteristics information

#### Development tools

Qt framework version 5.3.1 [20] was used to design the graphic user interface of the application. Qt was chosen because it is an open-source cross-platform application framework that can run various software and hardware platforms with little or no change in the source code while having the power and speed of a native application.

Visualization Toolkit (VTK) version 6.1.0 [21] was used to visualize the 3D human facial image with landmarks. VTK is an open-source cross-platform software system for 3D computer graphics visualization. VTK consists of a C++ class library and it supports a wide variety of visualization algorithms such as scalar, vector, tensor, texture, and volumetric methods. It also supports advanced modelling techniques such as implicit modelling, polygon reduction, mesh smoothing, cutting, contouring, and Delaunay triangulation.

CMake version 3.0.2 [22] was used to generate Makefiles and workspaces that locate and include the Qt framework library and VTK library as built libraries for Microsoft Visual C++ (MSVC) compiler for compilation during the building process. CMake is necessary because VTK is an open-source project that requires a cross-platform build environment. Besides that, VTK is only available in source code format. In the process of building a VTK binary, CMake was used to configure and generate build systems such as Makefiles and Visual Studio project files automatically.

Qt Creator version 3.1.2 [23] is an integrated development environment (IDE) used to create source codes, build executable applications, and debug the applications. Qt Creator was chosen because it is part of a software development kit (SDK) for the Qt framework. Moreover, Qt Creator has built-in tools that support CMake wizard.

#### Locating landmarks automatically

**Fig. 5:**
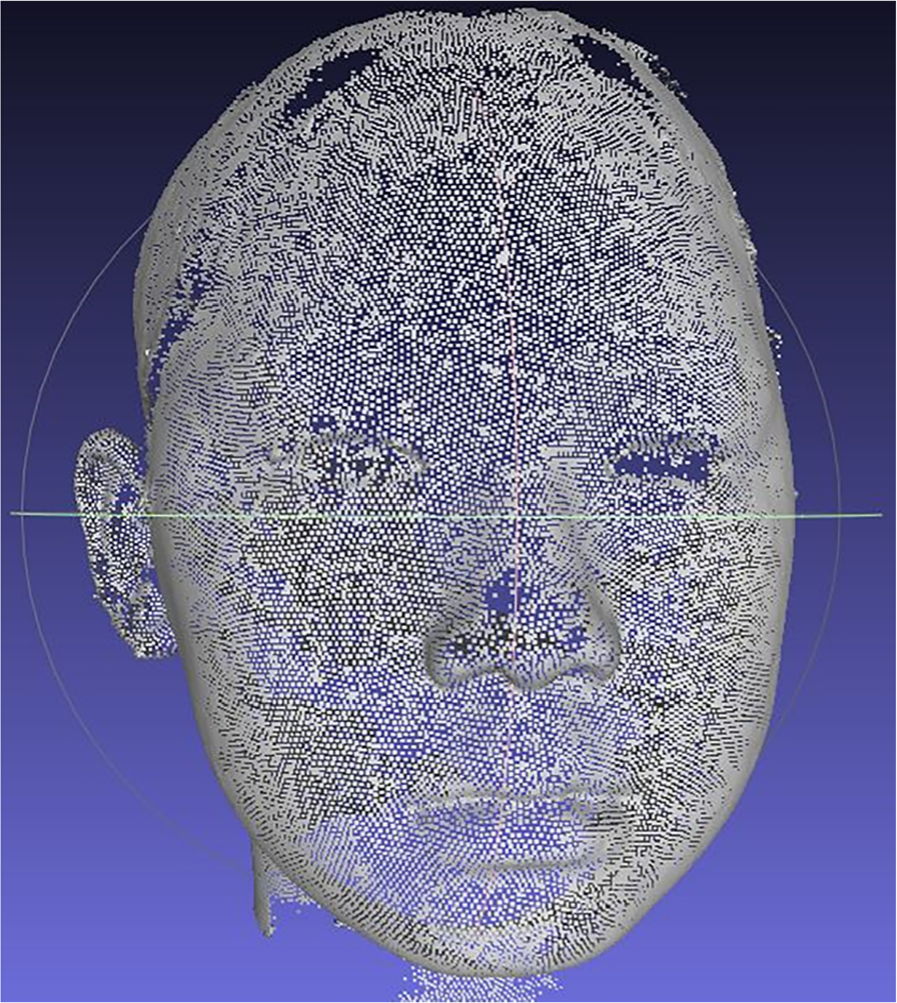
Vertices of a 3D facial image

A 3D image consists of a set of vertices. Vertices are defined by the *x*, *y*, and *z* coordinates in a 3D Euclidean space. Figure 5 shows all vertices of a 3D facial image. The surface is formed by forming a triangle mesh using the three vertices.

The 3D facial images were represented in a Wavefront OBJ file format. The Wavefront OBJ format represents polygonal data in text form, and files were stored with the extension .obj.

An OBJ file contains several types of definitions. Lines beginning with a hash character (#) were comments. Lines beginning with the letter “v” were vertices of geometric positions in space, followed by the first vertex listed in the file as index 1, and the subsequent vertices were numbered sequentially. Lines beginning with the letters “vn” were normal, followed by the first normal in the file labelled as index 1, and the subsequent normal were numbered sequentially. Lines beginning with the letters “vt” were texture coordinates. The first texture coordinated in the file was labelled as index 1, and the subsequent textures were numbered sequentially. Lines beginning with the letter “f” were polygonal faces. The numbers are indices of the arrays of vertex positions, texture coordinates, and normal respectively.

#### Pronasale (*prn*)

**Fig. 6:**
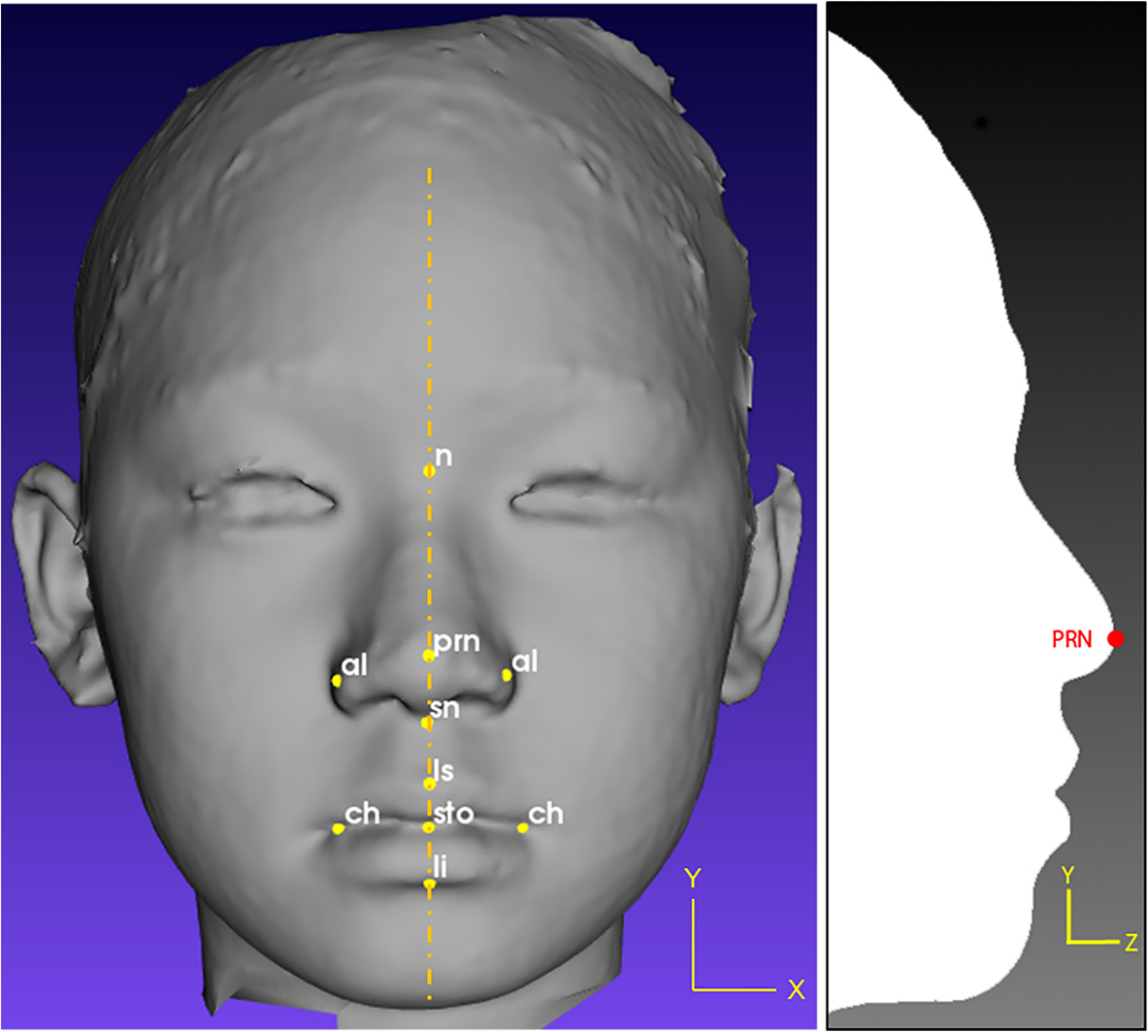
Locating the *prn* landmark

Regular expression was used to identify lines starting with the letter “v”. Then, the vertex with *x*, *y*, *z* coordinates was extracted and stored in the vertices list. After obtaining the coordinates from the list of vertices, the maximum *z* coordinate value was searched. The vertex with the maximum *z* coordinate was assigned as *prn* as shown in Fig. 6.

#### Nasion (*n*)

**Fig. 7:**
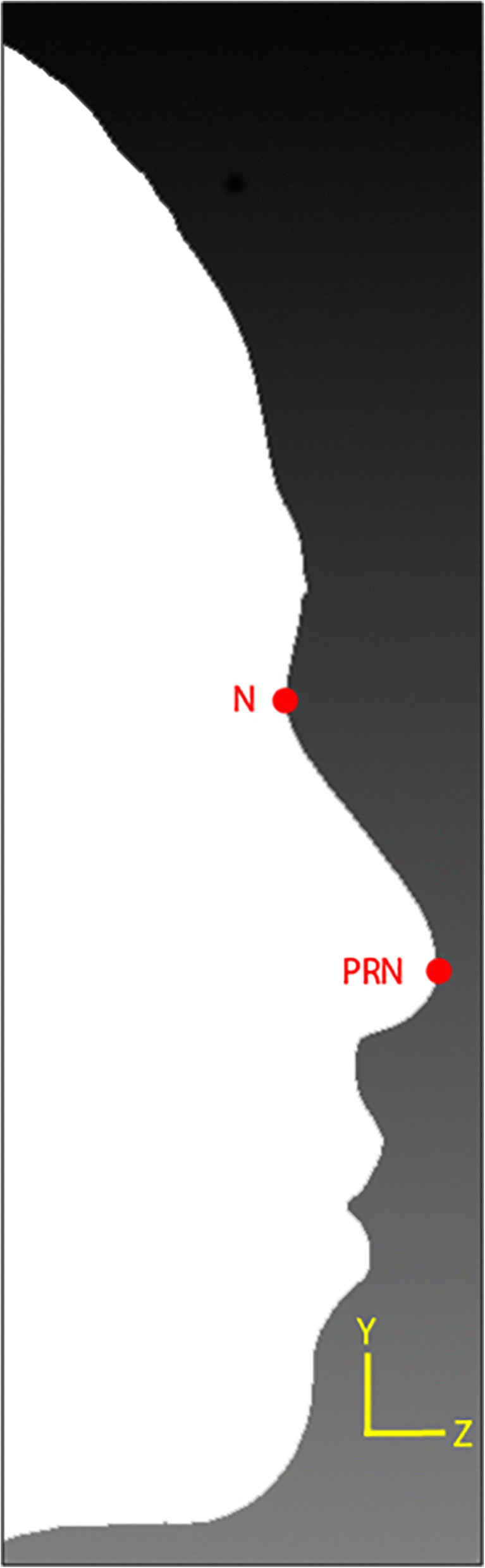
Locating the *n* landmark

Once the location of *prn* was obtained, a local *z* minimum value was searched along the *y*-axis located at *prn*’s *x* coordinate for *y* value larger than *prn y* coordinate. The vertex with said local *z* minimum value was assigned as *n* as shown in Fig. 7.

#### Subnasale (*sn*)

**Fig. 8:**
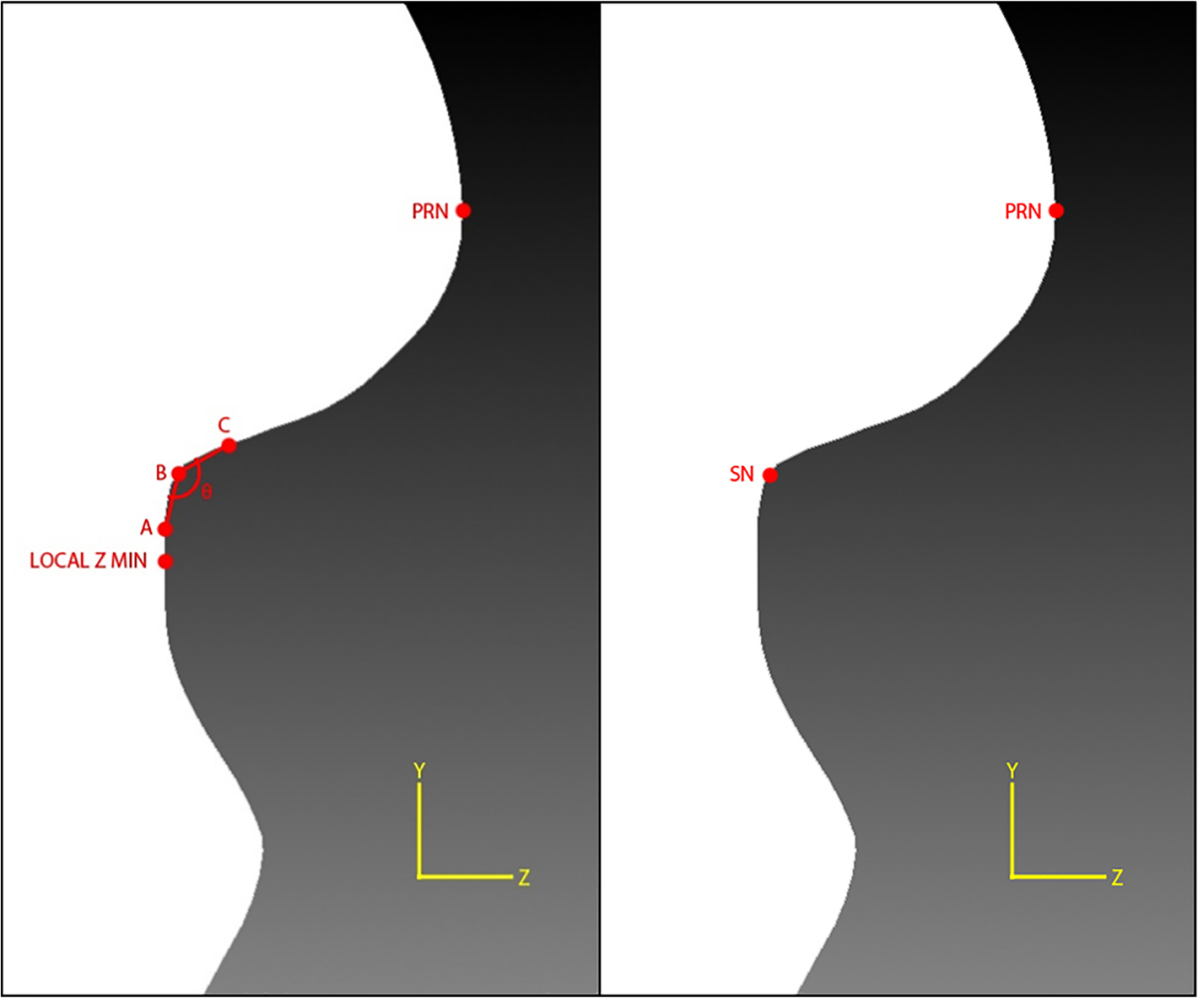
Locating the *sn* landmark

Then, another local *z* minimum value was searched along the same *y*-axis located at the *x* coordinate for a *y* value smaller than the *prn y* coordinate using the following equations. After obtaining the vertex with said local *z* minimum value, the angle between three vertices was calculated from that vertex upwards along the same *y*-axis located at *prn x* coordinate. The vertex with smallest angle was assigned as *sn* as shown in Fig. 8.

$$ 3\  vertices\ A\left(x,y,z\right),B\left(x,y,z\right), and\ C\left(x,y,z\right) $$

$$ vector\ \overrightarrow{BA}=\left({A}_x-{B}_x,{A}_y-{B}_y,{A}_z-{B}_z\right) $$

$$ vector\overrightarrow{\  BC}=\left({C}_x-{B}_x,{C}_y-{B}_y,{C}_z-{B}_z\right) $$

$$ Magnitude\ \left\Vert \overrightarrow{BA}\right\Vert =\sqrt{{\left({A}_x-{B}_x\right)}^2+{\left({A}_y-{B}_y\right)}^2+{\left({A}_z-{B}_z\right)}^2} $$

$$ Magnitude\ \left\Vert \overrightarrow{BC}\right\Vert =\sqrt{{\left({C}_x-{B}_x\right)}^2+{\left({C}_y-{B}_y\right)}^2+{\left({C}_z-{B}_z\right)}^2} $$

$$ Normalize\ vector\ \widehat{BA}=\left(\frac{A_x-{B}_x}{\left\Vert \overrightarrow{BA}\right\Vert },\frac{A_y-{B}_y}{\left\Vert \overrightarrow{BA}\right\Vert },\frac{A_z-{B}_z}{\left\Vert \overrightarrow{BA}\right\Vert }\ \right) $$

$$ Normalize\ vector\ \widehat{BC}=\left(\frac{C_x-{B}_x}{\left\Vert \overrightarrow{BC}\right\Vert },\frac{C_y-{B}_y}{\left\Vert \overrightarrow{BC}\right\Vert },\frac{C_z-{B}_z}{\left\Vert \overrightarrow{BC}\right\Vert }\ \right) $$

$$ \overrightarrow{BA}\cdotp \overrightarrow{BC}=\frac{A_x-{B}_x}{\left\Vert \overrightarrow{BA}\right\Vert}\times \frac{C_x-{B}_x}{\left\Vert \overrightarrow{BC}\right\Vert }+\frac{A_y-{B}_y}{\left\Vert \overrightarrow{BA}\right\Vert}\times \frac{C_y-{B}_y}{\left\Vert \overrightarrow{BC}\right\Vert }+\frac{A_z-{B}_z}{\left\Vert \overrightarrow{BA}\right\Vert}\times \frac{C_z-{B}_z}{\left\Vert \overrightarrow{BC}\right\Vert } $$

$$ \measuredangle ABC={\cos}^{-1}\left(\overrightarrow{BA}\cdotp \overrightarrow{BC}\right) $$

#### Labiale superius (*ls*) and stomion (*sto*)

**Fig. 9:**
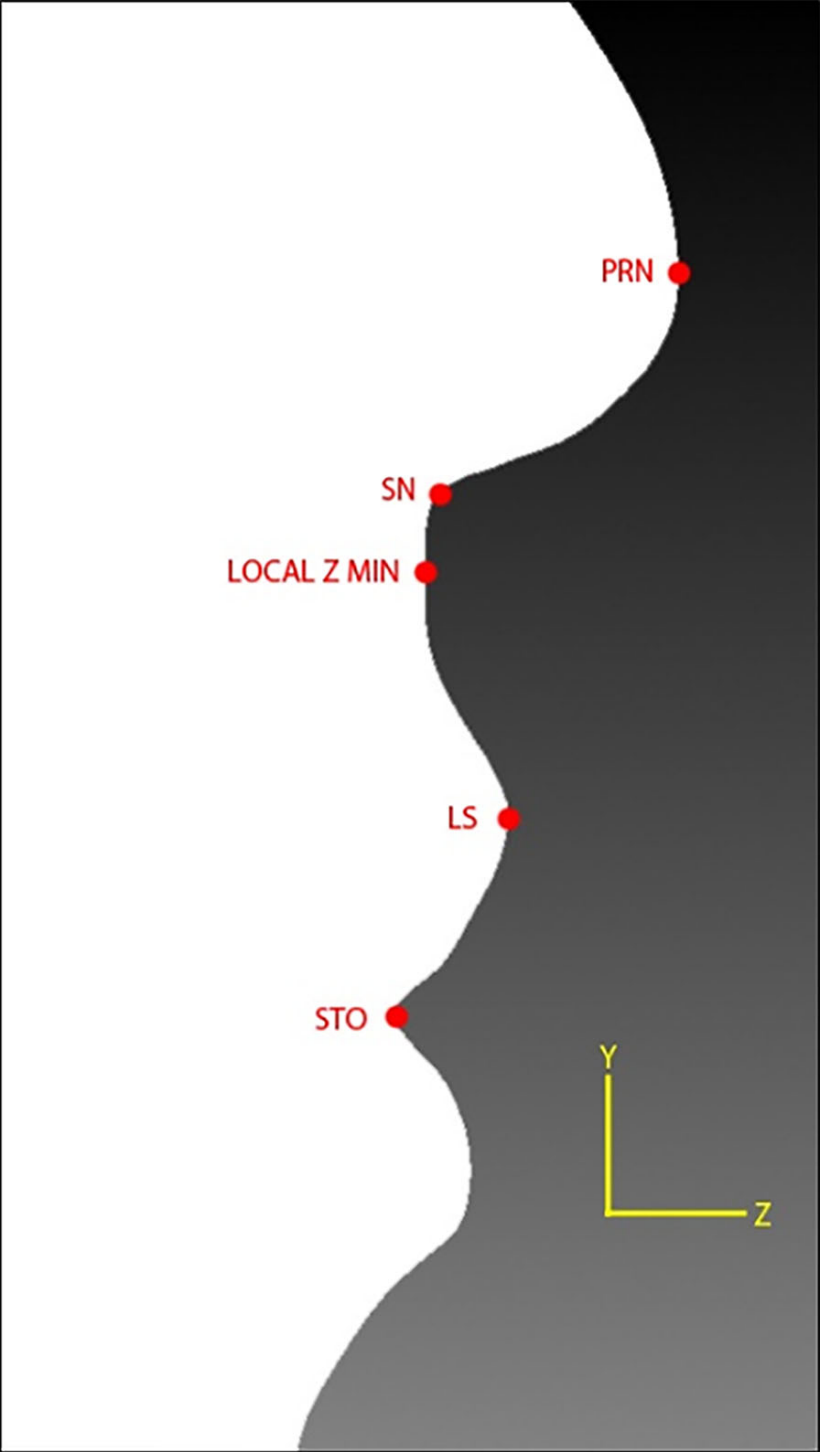
Locating the *ls* and *sto* landmarks

Next, from the local *z* minimum, a local *z* maximum value was searched downward the same *y*-axis located at *prn x* coordinate. The vertex with said local *z* maximum value was assigned as *ls*. From *ls*, a local *z* minimum value was searched downward and the vertex with said local *z* minimum value was assigned as *sto* as shown in Fig. 9.

#### Labiale inferius (*li*)

**Fig. 10:**
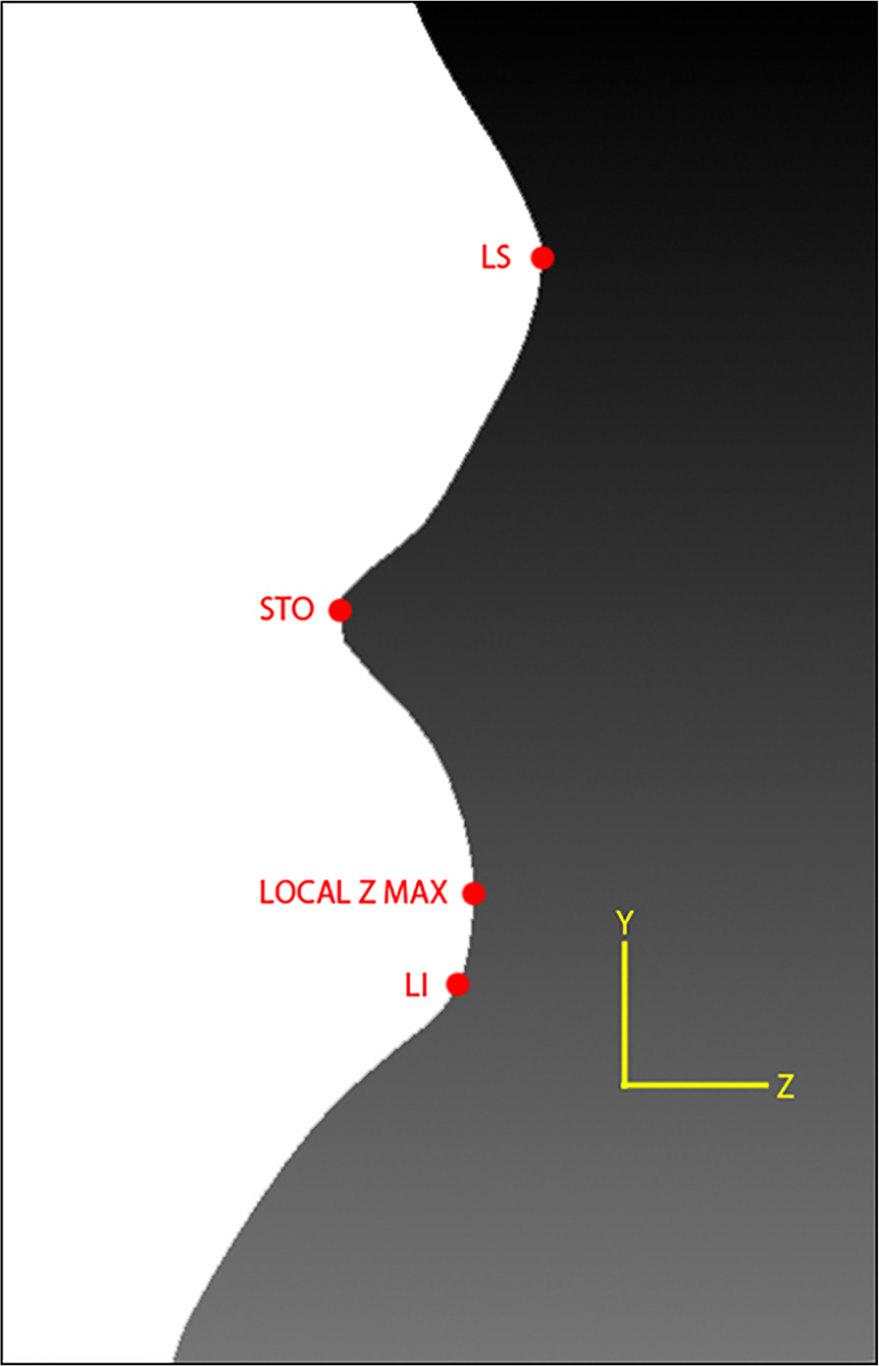
Locating the *li* landmark

Once *sto* was obtained, another local *z* maximum value was searched downward and found by using the same method for obtaining *sn*. The angle between the three vertices was calculated downward using the above equations. The vertex with the smallest angle was then assigned as *li* as shown in Fig. 10.

#### Alare (*al*)

**Fig. 11:**
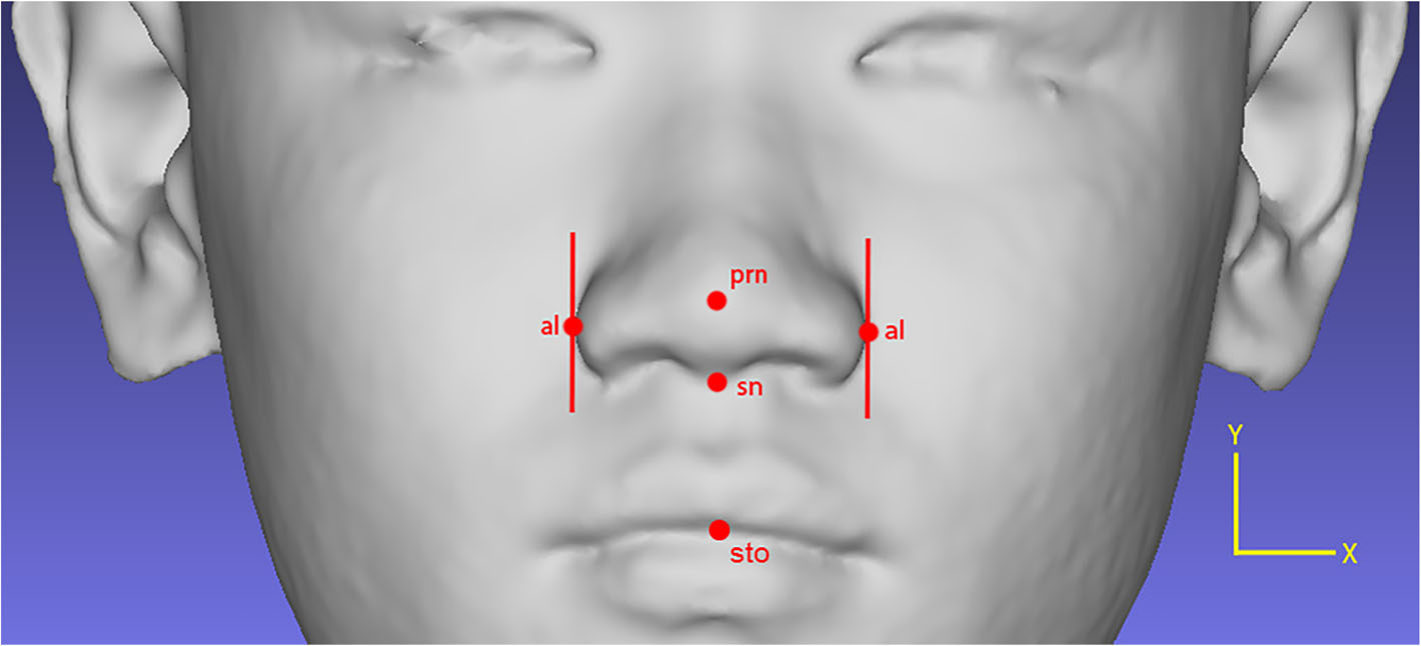
Locating the *al* landmark

Based on the two landmarks, *prn* and *sn*, the vertices in which *y* and *z* coordinate values lie between *prn* and *sn* were sought. From the vertices found, the vertex with the smallest *x* coordinate value was assigned as *al* on the left while the vertex with the largest *x* coordinate value was assigned as *al* on the right as shown in Fig. 11.

#### Chelion (*ch*)

**Fig. 12:**
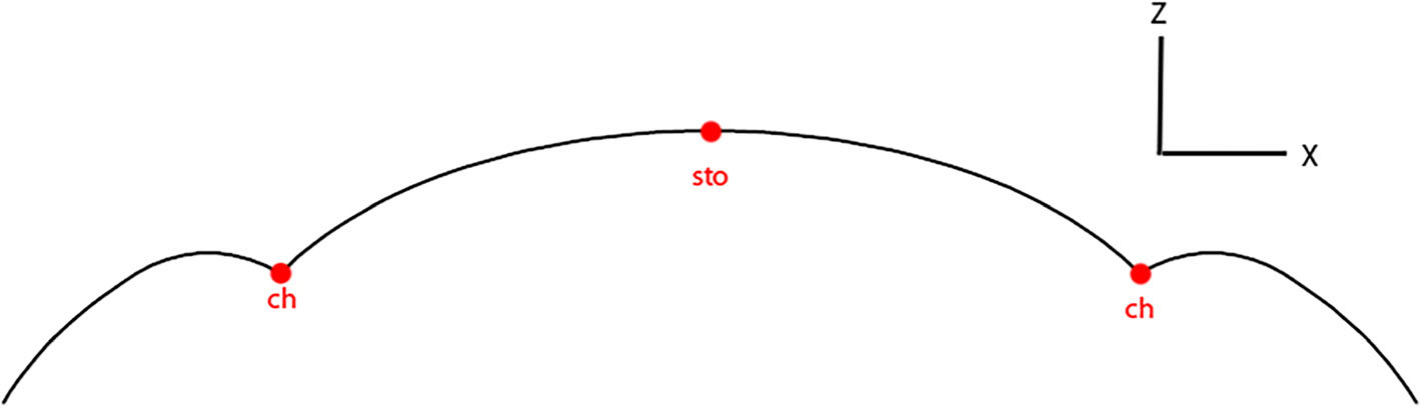
Locating the *ch* landmark

After obtaining *sto*, the angle between the three vertices along the *x*-axis located at *sto y* coordinate value was calculated. The vertex with the smallest angle value to the left of *sto* was assigned as *ch* on the left while the vertex with the smallest angle value to the right of *sto* was assigned as *ch* on the right as shown in Fig. 12.

### Extracting linear measurements and computing proportional indices

Once all landmarks were detected on the 3D facial image, the distance between the landmarks for all eight linear measurements as mentioned in Table 1 was calculated by using the Euclidean distance functions. In three-dimensional Euclidean spaces, if p = (p1, p2, p3) and q = (q1, q2, q3), then the distance is given by the following equation.

$$ d\left(p,q\right)=\sqrt{{\left({p}_1-{q}_1\right)}^2+{\left({p}_2-{q}_2\right)}^2+{\left({p}_3-{q}_3\right)}^2} $$

The five proportional indices shown in Table 2 above were calculated according to the ratio of linear measurements.

### Indirect anthropometry

#### Plotting the craniofacial landmarks manually

**Fig. 13:**
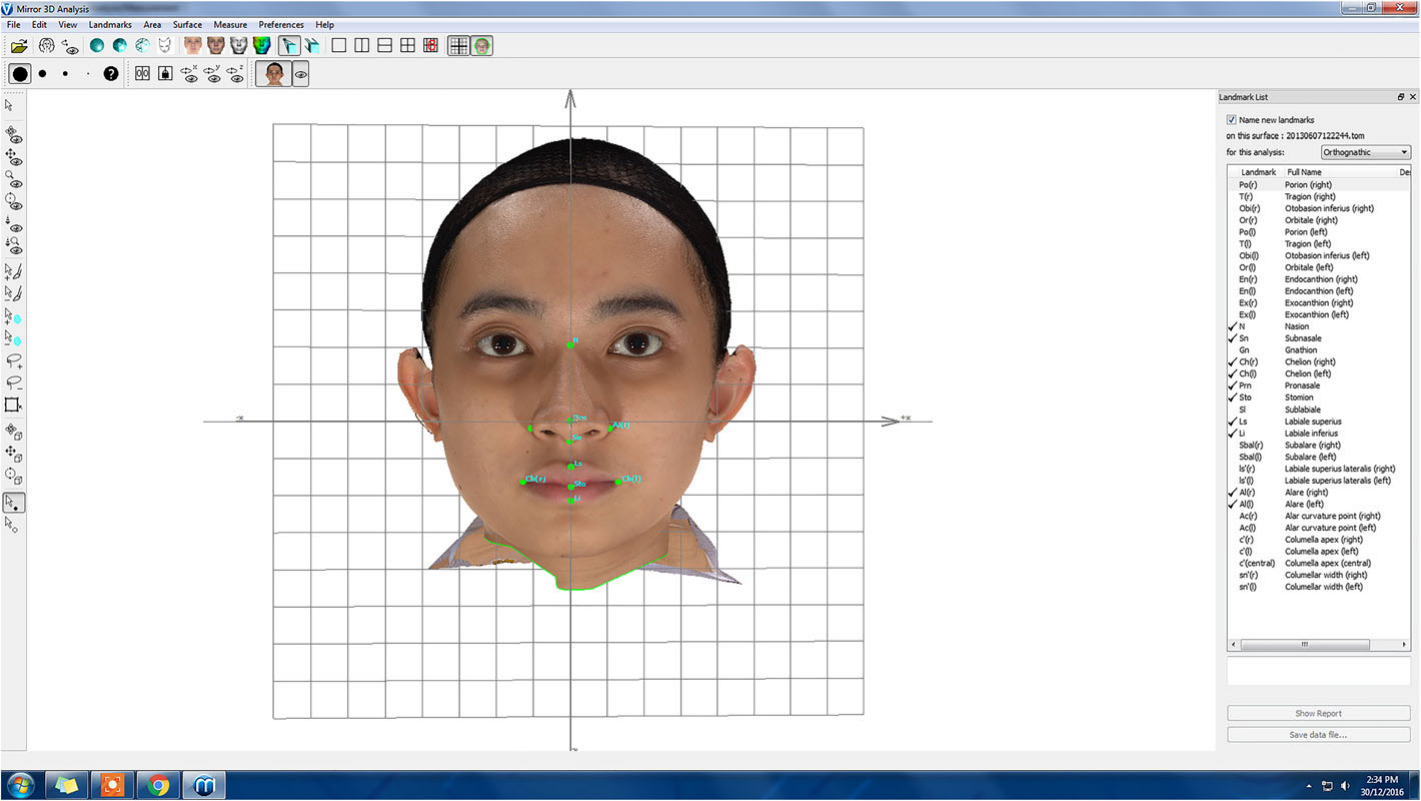
Annotated landmarks on a 3D facial image by an examiner using the Mirror software

The ten landmarks were located manually by a dedicated examiner on a 3D facial image using the Mirror software as shown in Fig. 13. The positions of the landmarks were identified first and several steps were then taken in order to familiarize with the positions. Once the 3D facial image was uploaded, a frontal view of the 3D facial image was displayed. The 3D facial image position needed to be symmetrized on both sides and it was adjusted based on the grid-axis. Once the 3D image was symmetrized, all landmarks were plotted at the respective positions.

#### Extracting linear measurements and computing proportional indices

**Fig. 14:**
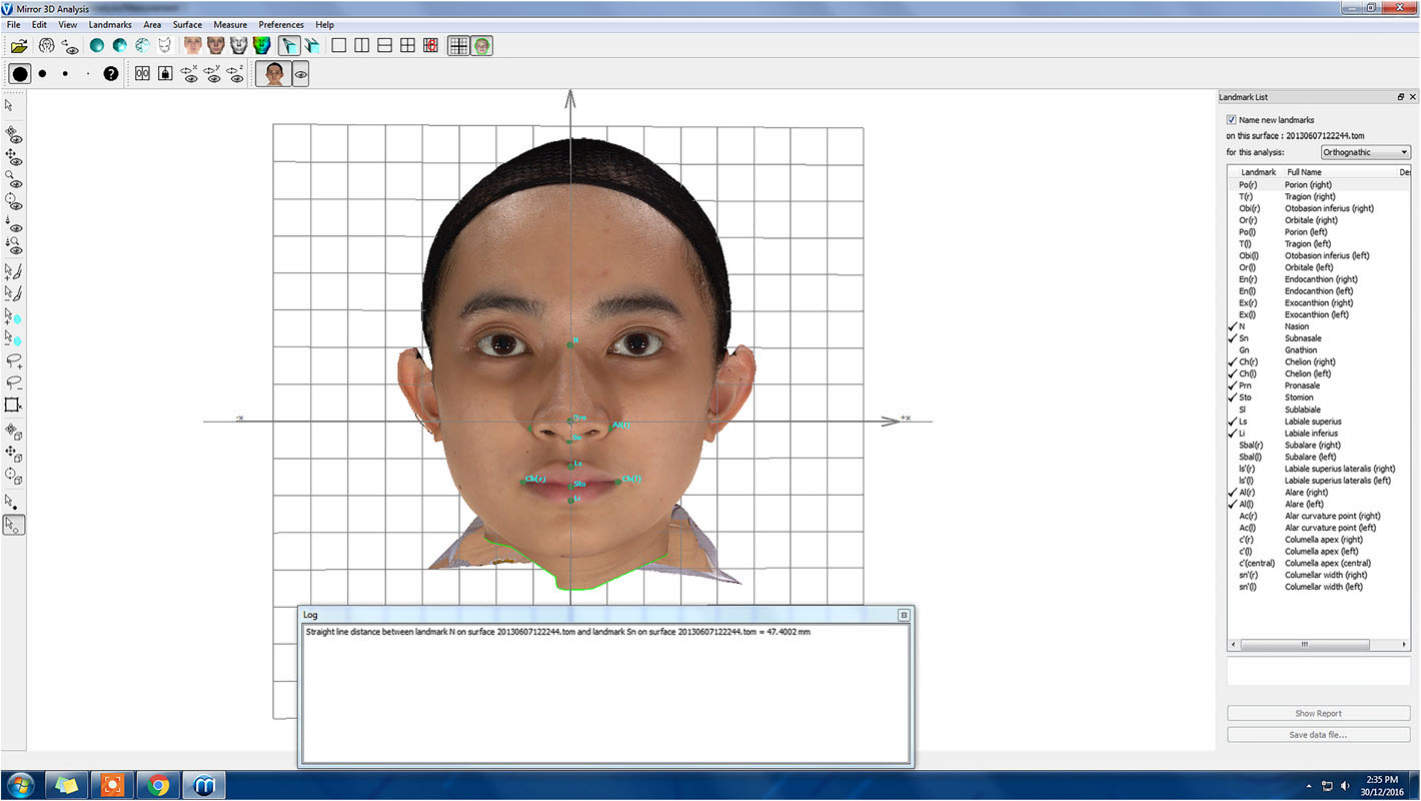
Linear measurements extraction using the Mirror software

Once the plotting of all landmarks was completed, the linear measurements between the landmarks were calculated by using the built-in function in the Mirror software named ‘Distance and Straight Line Between Landmarks’. The linear measurements were then displayed in a window at the bottom of the image as shown in Fig. 14. These linear measures were used to compute the proportional indices shown in Table 2.

### System evaluation

The term validity is generally used to describe whether the measured data accurately reflects an underlying but intangible construct [24]. Meanwhile, reliability is the degree to which an assessment tool produces stable and consistent results [25]. Intraclass correlation (ICC) is a general measurement of agreement or consensus, where the measurements used are assumed to be parametric (continuous and has a Normal distribution). The Coefficient represents an agreement between two or more raters or evaluation methods on the same set of subjects [26].

Statistical data analysis was performed using SPSS version 23 to determine the reliability of linear measurements that were taken manually in IA method through an ICC test and to evaluate the validity of the ACL system by comparing the two methods mentioned above through a Paired t-test. The ICC test was performed to check the reliability of the data taken manually by the examiners. It was also performed to check the chances of obtaining repeatable measurement readings. Thus, if the data has high reliability which is *p* > 0.7, it could be used as statistical data. Normality test was performed to check whether the recorded result data were normally distributed or not. If it is normally distributed (significant value, *p* > 0.05), the data could be used in other statistical tests such as the Paired t-test. If it is not normally distributed, the data could only be used in non-parametric tests. Paired t-test was performed to compare the means of the results. It is important in differentiating the mean difference between the two different readings.

## Results & discussion

### Reliability of the manual readings from IA

Table 3 shows the results of ICC tests from the reliability analysis of manual readings of female and male subjects. The estimation of reliability of linear measurements between female subjects is only 0.96, with a 95% confidence interval, CI (0.89, 0.99); while the estimation of reliability of linear measurements between male subjects is only 0.98, with a 95% confidence interval, CI (0.96, 0.99). The estimation of reliability of linear measurements between both female and male subjects is 0.99, with a 95% confidence interval, CI (0.98, 0.99). These values provide enough evidence to support the reliability of the linear measurements between the subjects and is almost a perfect agreement since ICC value is more than 0.7 (> 0.7), in the range of 0.7–1.0. The results show that linear measurement values of male and female subjects that were taken manually have high consistency and reliability to each other and are valid to run in statistical analyses.

**Table 3:** ICC test results on manual readings of female and male subjects

Subject	Intra-class Correlation	95% Confidence Interval (CI)		Sig
		Lower Bound	Upper Bound	
female reading only	0.96	0.89	0.99	0.00
male reading only	0.98	0.96	0.99	0.00
All female & male reading	0.99	0.98	0.99	0.00

### Validity of the ACL

The validity of the landmark locations in ACL was performed by calculating the average distances of the landmarks as well as comparing it with a previous study, Liang et al. [17]. Furthermore, a paired t-test was performed to check the validity of the ACL (i) between the subjects and (ii) between the two methods, by comparing the linear measurements extracted from both ACL and IA. Tests were performed on female subjects only, male subjects only and a combination of both subjects. Formulated hypotheses shown below were tested using a 2-tailed paired t-test with the mean difference, μ > 0 and significance value, *p* = 0.05.

$$ Null\ hypothesis:{\mu}_1-{\mu}_2<0,p>0.05 $$

$$ Alternative\ hypothesis:{\mu}_1-{\mu}_2>0,p<0.05 $$

$$ where\ {\mu}_1 is\  ACL\  and\ {\mu}_2\  is\  IA $$

#### Validity of the landmark locations

In order to validate the ACL, the landmark locations were compared with the landmarks manually plotted by the examiner as proposed by Liang et al. As shown in Table 4, the average distances of the eight linear measurements generated by the ACL to the IA were 2.16 mm. Compared to the results of Liang et al., the average distances of the landmarks were 2.23 mm. Despite the datasets being different between this study and Liang et al., we were able to demonstrate the overall performance of this study.

**Table 4:** Average distances (mm) and standard deviations of our method compared with Liang et al. [17]

Landmark	Our method	Liang et al. [17]
*n-sn*	1.44 ± 2.43	2.92 ± 1.62
*alR-alL*	−0.83 ± 1.90	1.78 ± 1.15
*sn-prn*	1.22 ± 1.55	1.59 ± 0.81
*chR-chL*	−0.56 ± 3.68	3.08 ± 2.14
*sn-sto*	5.79 ± 1.42	2.45 ± 0.80
*ls-sto*	3.51 ± 0.91	1.49 ± 0.90
*sn-ls*	3.49 ± 1.64	2.27 ± 1.15
*sto-li*	3.23 ± 1.44	2.27 ± 1.41

There are 27 landmarks listed by Liang et al. However, sellion (*se*), right and left alar curvature (*ac*), sublabiale (*sl*), right and left subalare (*sbal*), right and left crista philtra (*cph*), ganthion (*gn*), right and left endocanthion (*en*), right and left exocanthion (*ex*), right and left superaurale (*sa*), right and left postaurale (*pa*) were not considered in this study. Moreover, in this study, we focused more on the eight inter-landmarks distances to get the linear measurement rather than merely the location of the landmarks themselves.

#### Validity of the ACL against IA on between the subjects

As shown in Table 5, for the 30 female subjects only, the results for *n*-*sn*, *al*-*al*, and *ch*-*ch* with *p*-values of 0.21, 0.11 and 0.66 respectively were non-significant. As for the 30 male subjects only, the results for *al*-*al*, *sn*-*prn*, and *ch*-*ch* with p-values of 0.10, 0.06 and 0.49 respectively were non-significant. However, when both 60 subjects were combined, non-significance is only shown for *ch*-*ch* with a p-value of 0.41. As for the other six linear measurements, namely *n*-*sn*, *sn*-*prn*, *sn*-*sto*, *ls*-*sto*, *sn*-*ls* and *sto*-*li*, have shown a very statistically significant result where *p* = 0.00, where p is less than 0.05. One linear measurement in particular, *al*-*al*, shows an acceptable significant result where *p* = 0.03, where p is still less than 0.05.

**Table 5:** Paired t-test results on the validity of the automated craniofacial landmarks between the subjects

Linear measurements	30 female subjects only		30 male subjects only		All 60 female & male subjects	
	t-test	*p*-value	t-test	*p*-value	t-test	*p*-value
n-sn	1.28	0.21	3.93	0.00	3.25	0.00
al-al	−1.63	0.11	−1.70	0.10	−2.33	0.03
sn-prn	3.56	0.00	2.00	0.06	4.31	0.00
ch-ch	−0.44	0.66	−0.71	0.49	−0.84	0.41
sn-sto	12.82	0.00	18.04	0.00	22.19	0.00
ls-sto	16.50	0.00	15.18	0.00	21.10	0.00
sn-ls	7.54	0.00	10.36	0.00	11.65	0.00
sto-li	6.69	0.00	8.22	0.00	12.23	0.00

Çeliktutan et al. [27] mentioned that the variability of landmark measurement was interfered with by intrinsic and extrinsic factors. Landmark appearances can differ through intrinsic factors such as facial structure variability while the extrinsic factor interferes with the landmark distance through different facial expressions and poses such as smiling. Zhang et al. [28] mentioned that smiling attributed in interferences and the commonly affected areas are the area around the nose and the two corners of the mouth. The uses of automated land-marking algorithms have worked well for intrinsic factors so far but there is no guarantee for extrinsic factors. This limitation is present in this study whereby both for female subjects only and male subjects only, the results were non-significant for *al*-*al* and *ch*-*ch* which are located at the nose and the two corners of the mouth. The other non-significant findings were the *n*-*sn* for female subjects only and the *sn*-*prn* for male subjects only. As mentioned in Othman et al. [29] there was difficulty in placing landmarks on *n* as that may affect the distance of *n*-*sn* due to the variability of the nose curvature area.

In addition, the present work found a positive relationship between the ACL and IA whereby the paired t-test shows positive values on *n*-*sn*, *sn*-*prn*, *sn*-*sto*, *ls*-*sto*, *sn*-*ls* and *sto*-*li*, which indicated that positive values are more than the negative values found on *al*-*al* and *ch*-*ch*. It shows that the ACL is more accurate compared to the IA. Moreover, out of the *p*-values of the eight linear measurements, seven of them are statistically significant which supports the Alternative hypothesis and Rejects the null. Thus, there was enough strong evidence to show that the validity of ACL is better than IA.

#### Validity of the ACL against IA on between the methods

Tests to check the validity of the ACL against AI between the systems were performed by using the average of all eight linear measurements, extracted from both methods.

Since the number of samples for this work is only 60 subjects for both female and male and is less than 100 samples, a Normality test was performed to check the distribution of the recorded data. Table 6 shows the results of the normality test on the eight linear measurements using Shapiro-Wilk’s test. All the significant values* are more than 0.05 (*p* > 0.05). Therefore, the eight linear measurements were assumed to be approximately normally distributed in terms of Shapiro-Wilk’s test and were processed and analyzed through a paired t-test.

**Table 6:** Normality test based on Shapiro-Wilk’s test

Subjects	Shapiro-Wilk		
	Statistic	Df	Sig.
ACL - female	.892	8	.245*
IA - female	.864	8	.132*
Difference_female	.954	8	.752*
ACL- male	.892	8	.244*
IA - male	.861	8	.124*
Difference_male	.937	8	.584*
ACL - female & male	.892	8	.246*
IA - female & male	.863	8	.128*
Difference_female & male	.943	8	.640*

Based on the sample statistics of female subjects shown in Table 7, the mean of ACL is 23.86 while IA is 21.77. It shows that the mean of ACL for eight different linear measurements is higher than the mean of IA and that there is a positive mean difference. As shown in Table 8, the mean difference between the ACL and IA is 2.09 in which μ1 - μ2 > 0, the standard deviation is 2.13 and the standard error of mean is 0.75. The t value is 2.78 with 7 degrees of freedom while the *p*-value is 0.027 ≈ 0.03.

**Table 7:** Paired sample statistics for female subjects

		Mean	N	Std. Deviation	Std. Error Mean
Pair 1	ACL - female	23.8588	8	14.52389	5.13497
	IA - female	21.7675	8	16.00595	5.65896

**Table 8:** Paired t-test for female subjects

		Paired Differences					t	df	Sig. (2-tailed)
		Mean	Std. Deviation	Std. Error Mean	95% Confidence Interval of the Difference				
					Lower	Upper			
Pair 1	ACL - IA	2.09125	2.12503	.75131	.31468	3.86782	2.783	7	.027

Table 9 shows the sample statistics of male subjects where the mean of ACL is 25.53 and IA is 23.30. It shows that the mean of ACL for eight different linear measurements is higher than the mean of IA and that there is a positive mean difference. As shown in Table 10, the mean difference between the ACL and IA is 2.24 in which μ1 - μ2 > 0, while the standard deviation is 2.40 and the standard error of mean is 0.85. The t value is 2.64 with 7 degrees of freedom while the p-value is 0.034 ≈ 0.03.

**Table 9:** Paired sample statistics for male subjects

		Mean	N	Std. Deviation	Std. Error Mean
Pair 1	ACL - male	25.5313	8	15.40150	5.44525
	IA - male	23.2950	8	16.88135	5.96846

**Table 10:** Paired t-test for male subjects

		Paired Differences					t	df	Sig. (2-tailed)
		Mean	Std. Deviation	Std. Error Mean	95% Confidence Interval of the Difference				
					Lower	Upper			
Pair 1	ACL - IA	2.23625	2.40082	.84882	.22911	4.24339	2.635	7	.034

The results in Table 11 show that the mean of ACL and IA for sample statistics of a combination of female and male subjects have values of 24.70 and 22.53 respectively. It also shows that the mean of ACL for eight different linear measurements is higher than the mean of IA and that there is a positive mean difference. Table 12 shows that the mean difference between the ACL and IA is 2.16 in which μ1 - μ2 > 0, while the standard deviation is 2.25 and the standard error of mean is ≈0.80. The t value is 2.71 with 7 degrees of freedom while the p-value is 0.03.

**Table 11:** Paired sample statistics for ACL and IA of a combination of female & male subjects

		Mean	N	Std. Deviation	Std. Error Mean
Pair 1	ACL	24.6963	8	14.96077	5.28943
	IA	22.5325	8	16.44085	5.81272

**Table 12:** Paired t-test for ACL and IA for the combination of female & male subjects

		Paired Differences					t	df	Sig. (2-tailed)
		Mean	Std. Deviation	Std. Error Mean	95% Confidence Interval of the Difference				
					Lower	Upper			
Pair 1	ACL - IA	2.16375	2.25230	.79631	.28078	4.04672	2.717	7	.030

All results show positive mean differences, in which μ1 - μ2 > 0, and significant values (*p* < 0.05) between ACL and IA. The accuracy between two different systems can be proven wherein μ1 - μ2 > 0, which shows that the ACL is more accurate compared to the IA. The Alternative hypothesis is accepted when the p ≈ 0.03 is less than 0.05 (p < 0.05) and the Null hypothesis is rejected.

### The ACL system

**Fig. 15:**
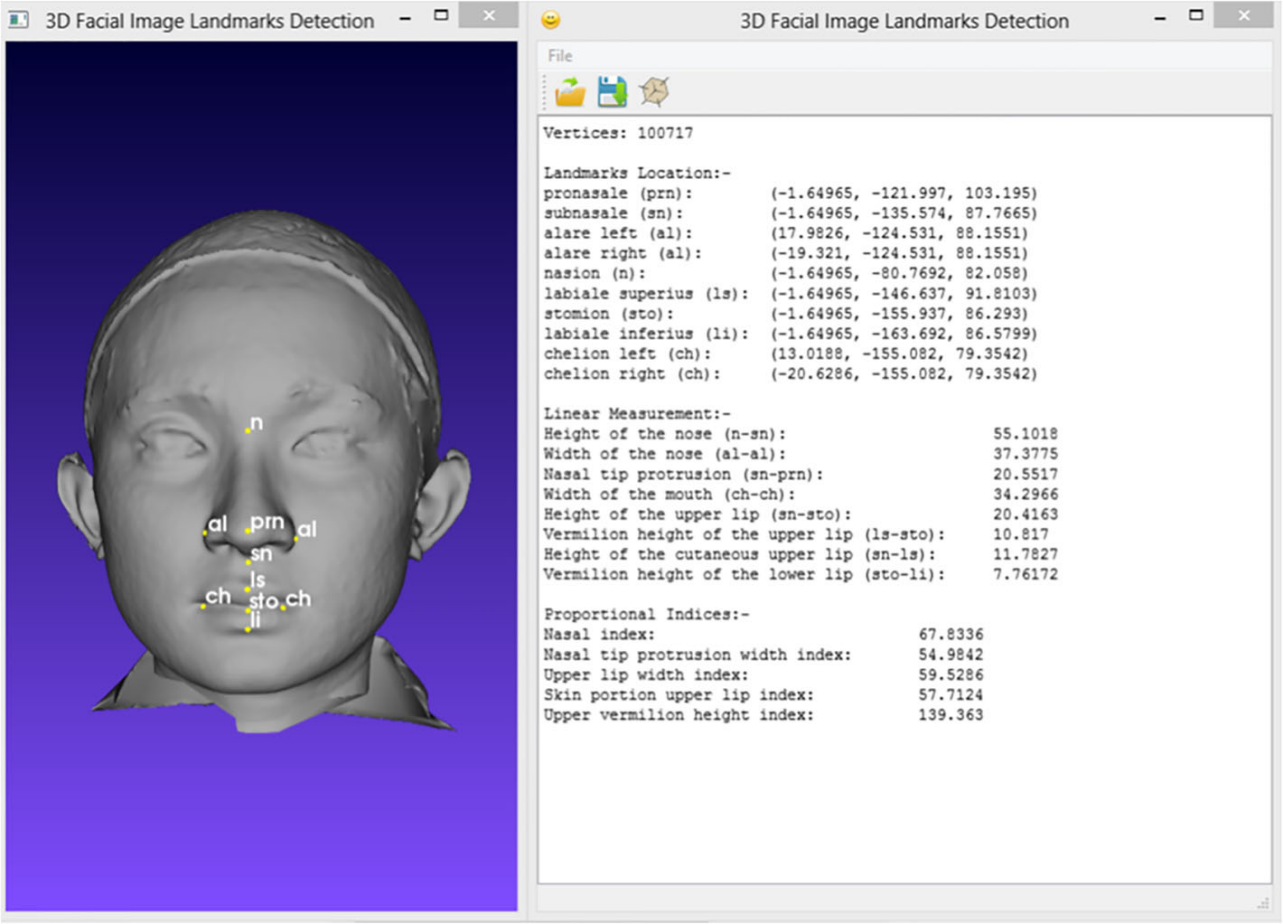
The ACL system

As a proof-of-principle that this approach could be applied to automatically detect the craniofacial landmarks using geometry characteristics information, a stand-alone system was developed as shown in Fig. 15. This system allows users to upload a 3D facial image as an unknown face, detect landmarks, extract linear measurements, and obtain the proportional indices of the 3D facial image.

Graphical User Interface (GUI) was built to complete the information extraction from the user’s side with just a few clicks. The GUI is even suitable for users with minimal knowledge as the system will detect the craniofacial landmarks and extract the information automatically. Not only will it result in a 3D facial image with annotated landmarks, users can also view information on the landmarks’ coordinates, linear measurements and proportional indices. As this information might be useful to experts for further data analysis, the results can be saved in a CSV file format.

The automated craniofacial landmarks coordinate is able to register landmarks on 3D mesh facial images and obtain measurements automatically. In addition, no error caused by human bias will happen because craniofacial landmarks’ registration is performed by the same computer algorithm. Consequently, time consumption and accuracy has been improved compared to when obtaining the measurements manually. Furthermore, no trained personnel were required to perform this task. Anyone with basic computing skills should be able to use the system easily. Therefore, resources such as time and costs of hiring well-trained examiners can be redirected towards craniofacial anthropometry studies instead of spending on obtaining the measurements data.

**Fig. 16:**
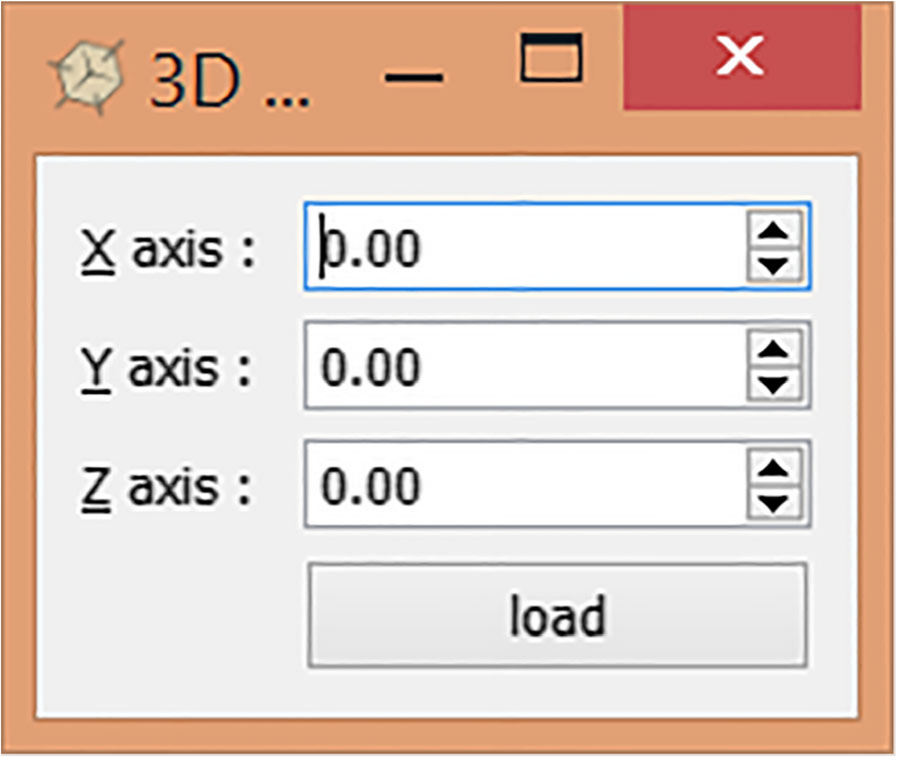
3D rotation for normalisation

However, the system does have limitations so that the usability testing with the end users could not be performed thus far. Input images for the system must be in the correct pose and orientation. Therefore, all the 3D facial images must undergo a pre-processing stage before it can be used in the system. In the pre-processing stage, 3D rotation is done manually on the 3D facial images to correct its orientation. An application was made to perform the 3D rotation as shown in Fig. 16, whereby three rotation matrices were used to rotate all vertices in the 3D mesh image for angle θ about the *x*-, *y*- or *z*-axis respectively.

Thus, it requires some time to manually rotate the 3D facial images. However, time consumption is still less than manually plotting the landmarks on 3D images.

Due to this limitation, future enhancement is necessary. To avoid the necessity of the 3D images pre-processing step, the system should be able to find landmarks accurately regardless of orientation. Deformable registration method is recommended for future system improvement, in which a reference 3D facial mesh model with landmarks is moving around a fixed target 3D image to search for the best alignment between the target and reference image. The moving mesh should be able to stretch, twist, compress and rotate during the searching process. The reference model and allowable degree of deform has to be determined by using a machine-learning method to find out the optimum solution for all faces. Deformable registration method is available in image processing software packages such as Insight Segmentation and Registration Toolkit (ITK), which is an open-source, cross-platform system that provides developers with an extensive suite of software tools for image analyses. With these few enhancements in the future, the usability of ACL system will be tested with the end users such as the clinicians and examiners.

## Conclusions

A system, named ACL, which is able to automatically locate eight craniofacial landmarks using geometry characteristics information and extract the linear measurements, was developed. This system is reliable because the validity testing shows that out of the *p*-values of the eight linear measurements, seven of them were statistically significant which supports the alternative hypothesis and rejects the null. ACL also provides a user-friendly interface and demonstrates the practicability to be used as another alternative tool for indirect anthropometry. It is free from human bias and can be done within a very short amount of time. This study can be expanded for other measurements such as volume and area.

## Abbreviations

ACL: Automated craniofacial landmarks
*alL*: Alare (left)
*alR*: Alare (right)
*chL*: Chelion (left)
*chR*: Chelion (right)
GUI: Graphical user interface
IA: Indirect anthropometry
*li*: Labiale inferius
*ls*: Labiale superius
*n*: Nasion
*prn*: Pronasale
*sn*: Subnasale
*sto*: Stomion
